# Frailty, nutrition-related parameters, and mortality across the adult age spectrum

**DOI:** 10.1186/s12916-018-1176-6

**Published:** 2018-10-26

**Authors:** Kulapong Jayanama, Olga Theou, Joanna M Blodgett, Leah Cahill, Kenneth Rockwood

**Affiliations:** 1 Chakri Naruebodindra Medical Institute, Faculty of Medicine Ramathibodi Hospital, Mahidol University, Bangkok, Thailand; 2 Department of Medicine, Dalhousie University, Halifax, Nova Scotia Canada; 3 Centre for Health Care of the Elderly, QEII Health Sciences Centre, Nova Scotia Health Authority, Halifax, Nova Scotia Canada; 4 MRC Unit for Lifelong Health and Ageing, UCL, London, UK; 5 Department of Nutrition, Harvard T.H. Chan School of Public Health, Boston, MA USA; 6 Division of Geriatric Medicine, Department of Medicine, Dalhousie University, Camp Hill Veterans’ Memorial Bldg., 5955 Veterans’ Memorial Lane, Halifax, Nova Scotia B3H 2E1 Canada

**Keywords:** Nutrition, Dietary intake, Frailty, Frailty index, Mortality, NHANES

## Abstract

**Background:**

Nutritional status and individual nutrients have been associated with frailty in older adults. The extent to which these associations hold in younger people, by type of malnutrition or grades of frailty, is unclear. Our objectives were to (1) evaluate the relationship between individual nutrition-related parameters and frailty, (2) investigate the association between individual nutrition-related parameters and mortality across frailty levels, and (3) examine whether combining nutrition-related parameters in an index predicts mortality risk across frailty levels.

**Methods:**

This observational study assembled 9030 participants aged ≥ 20 years from the 2003–2006 cohorts of the *National Health and Nutrition Examination Survey* who had complete frailty data. A 36-item frailty index (FI) was constructed excluding items related to nutritional status. We examined 62 nutrition-related parameters with established cut points: 34 nutrient intake items, 5 anthropometric measurements, and 23 relevant blood tests. The 41 nutrition-related parameters which were associated with frailty were combined into a nutrition index (NI). All-cause mortality data until 2011 were identified from death certificates.

**Results:**

All 5 anthropometric measurements, 21/23 blood tests, and 19/34 nutrient intake items were significantly related to frailty. Although most nutrition-related parameters were directly related to frailty, high alcohol consumption and high levels of serum alpha-carotene, beta-carotene, beta-cryptoxanthin, total cholesterol, and LDL-c were associated with lower frailty scores. Only low vitamin D was associated with increased mortality risk across all frailty levels. Seventeen nutrition-related parameters were associated with mortality in the 0.1–0.2 FI group, 11 in the 0.2–0.3 group, and 16 in the > 0.3 group. Overall, 393 (5.8%) of the participants had an NI score less than 0.1 (abnormality in ≤ 4 of the 41 parameters examined). Higher levels of NI were associated with higher mortality risk after adjusting for frailty and other covariates (HR per 0.1: 1.19 [95%CI 1.133–1.257]).

**Conclusions:**

Most nutrition-related parameters were correlated to frailty, but only low vitamin D was associated with higher risk for mortality across levels of frailty. As has been observed with other age-related phenomena, even though many nutrition-related parameters were not significantly associated with mortality individually, when combined in an index, they strongly predicted mortality risk.

## Background

Reflecting the increasing life expectancy of the global population [1], the number of adults aged 65 years or older is predicted to double by 2050 [2]. In parallel, the prevalence of age-related health deficits including cardiovascular, metabolic, cognitive, and musculoskeletal diseases is growing [3–6]. Frailty is a multiply determined, age-related state of vulnerability to adverse health outcomes compared with others of the same age [7, 8]. It is associated with a range of adverse outcomes, including morbidity, mortality, and increased healthcare costs [9, 10]. Frailty can be observed at all adult ages and is closely tied to ageing, suggesting that the prevalence of frailty is likely to increase as populations age [11]. Even so, two European cohorts have observed only very modest increases with age in the mean frailty, despite varying estimates in the extent of its lethality, especially in people with milder degrees of frailty [12, 13].

Against this background, two considerations motivate a more comprehensive understanding of the relationship between nutrition and frailty. First, the two are linked. The prevalence of malnourished individuals can be high in ageing populations, especially in rehabilitation, hospital, and nursing home settings [14, 15]. Malnutrition, which is affected by inadequate, excessive, or imbalance of energy or nutrient consumption, is associated with physical and cognitive impairment, poor quality of life, morbidity, and mortality in older individuals [16–20]. Malnutrition is also associated with higher levels of frailty [8, 21].

Second, optimal nutrition management can improve frailty [22, 23] and some nutrient intakes or supplements, for example, fish oil and antioxidants, are associated with reduced frailty levels [24–27]. Nutrition management therefore appears to make poor nutrition a modifiable risk factor in relation to frailty. Importantly too, nutrition management appears to work well, in both hospital and community settings, as part of multidimensional interventions that also include exercise, pharmacological treatment, and social support [28–31].

Despite these promising insights, the evidence about the relationship of nutrition-related parameters with frailty, and whether these associations hold in younger people and by type of malnutrition, is limited and inconsistent [32–35]. Further, the multiplicity of claims about which nutritional factors might be most important is a pragmatic obstacle to uptake [8, 36–38]. This obscures how the relationship might arise, and where new interventions might best be targeted. In other contexts in which the impact of age-related adverse outcomes varies by which items are studied, it has been useful to study deficits in the aggregate [39], something which has been variably applied in nutrition studies [40]. To help improve the understanding of the relationship between frailty and nutrition, this study aims (1) to evaluate the relationship between individual nutrition-related parameters and frailty, (2) to investigate the effect of these parameters on mortality risk across levels of frailty, and (3) to examine whether combining nutrition-related parameters in an index predicts mortality risk across frailty levels.

## Methods

### Study population and design

This observational study used data from 10,020 individuals aged 20 years or more from the 2003–2004 and 2005–2006 cohorts of the National Health and Nutrition Examination Survey (NHANES). NHANES is a series of publicly available, cross-sectional surveys focusing on the health and nutrition of non-institutionalized US residents [41, 42]. For the purpose of this study, 990 individuals with missing FI scores were excluded. The final sample included 9030 participants. Mortality status was identified from the death certificate records from the National Death Index in December 31, 2011, and survival time was counted from the date of the clinical examination to the death event.

Each participant signed written informed consent provided to participate. The NHANES protocol was approved by the institutional review board of the Centers for Disease Control and Prevention (CDC). As a matter of policy, our local Research Ethics Committee does not review secondary analyses of duly approved, publicly available data.

### Nutrition-related data

Of 84 nutrition-related parameters included in NHANES, 62 items had established cut points. Among them, 34 energy and nutrient intake items were estimated from dietary information recalled during the 24-h period prior to the interview. Five anthropometric measurements and 23 blood tests related to nutrition were collected with standard techniques. The normal range of each parameter is shown in Table 5 in Appendix. These cut points were taken from a standard textbook, the Dietary Reference Intake (DRIs), published guidelines, and previous studies [11, 43–55].

### Frailty index

The FI used in this study included 36 items and was modified from a previously validated FI in NHANES [11, 56] (Table 6 in Appendix). We excluded from the FI all items related to dietary intake or nutritional status (i.e. difficulty using fork and knife, difficulty preparing meals, glycohaemoglobin, triglyceride, creatinine, haemoglobin, mean corpuscular volume, total cholesterol, glucose, and sodium). The FI score, the number of deficits present divided by the total deficits considered, ranges between 0 and 1, and a higher score is associated with higher frailty. For stratification purposes, we grouped participants into 4 FI groups: FI ≤ 0.1 (fit), 0.1 < FI ≤ 0.2 (vulnerable), 0.2 < FI ≤ 0.3 (mildly frail), and FI > 0.3 (moderately/severely frail) [56].

### Nutrition index

A nutrition index (NI) was constructed following the deficit accumulation approach [57] by combining the 41 nutrition-related parameters that were related with higher frailty: counting the number of nutritional deficits in an individual and dividing by the total deficits considered. Low-density lipoprotein cholesterol (LDL-c) and subscapular skinfold were excluded from the NI due to high number of missing data: 53.9% for LDL-c and 23.8% for subscapular skinfold. Each nutritional parameter was scored “1” if the value fell outside the normal range and “0” otherwise. Abnormal values that were found to be protective for frailty (associated with lower levels of frailty) were also scored as 0 (Table 5 in Appendix). An NI score was only calculated for individuals with > 80% of the variables complete. The NI score ranges between 0 and 1; an NI score of 0 represents full nutritional health, while a score of 1 represents complete nutritional deficits. In the analysis, we used both the continuous NI score and a categorical variable: NI ≤ 0.2, 0.2 < NI ≤ 0.3, 0.3 < NI ≤ 0.4, 0.4 < NI ≤ 0.5, and NI > 0.5.

### Statistical analysis

Demographic characteristics of the subjects are presented as mean ± standard deviation (SD) for continuous variables and as frequency (%) for binary or categorical variables. All percentages and mean values were weighted using the sampling weights provided by NHANES. Multiple linear regression analysis was used to assess the associations between each nutrition-related parameter, NI and FI scores and is presented by *β*-coefficient with 95% confidence interval (CI). The mortality risk from each parameter across the FI group was analysed using Cox regression models, and the odds of mortality risk was presented using the hazard ratios and the associated 95%CI. All regression models were adjusted for potential covariates including age, sex, race, energy intake, educational level, marital status, employment status, smoking, and study cohort. Models which included energy, energy per weight, dietary fiber per energy intakes, and NI as predictors were not adjusted for energy intake. Annual household income was not included as covariate due to missing data. Statistical significance was considered as a *p* value < 0.05, and all reported probability tests were two-sided. The statistical analysis was conducted using IBM SPSS Statistics for Windows, Version 24.0. Armonk, NY: IBM Corp.

## Results

Of the 9030 included participants, 48% were male; their weighted mean age was 46.6 ± 16.9 years. When we stratified the sample by frailty, 5119 (56.7%), 2009 (22.2%), 1014 (11.2%), and 888 (9.8%) had an FI score < 0.1, 0.1–0.2, 0.2–0.3, and > 0.3, respectively. The weighted mortality rate was 6.5% (940/9030). The demographic characteristics of the sample by frailty categories are presented in Table 1. In the frailer groups, the mean age and number of people with female gender, lower education, non-full-time work, and low income were significantly higher (*p* < 0.001) (Table 1).

**Table 1:** Demographic characteristics of participants by frailty level

Characteristics	Frailty index score			
	≤ 0.1 *N* = 5119	> 0.1 to 0.2 *N* = 2009	> 0.2 to 0.3 *N* = 1014	> 0.3 *N* = 888
Age (year), mean ± SD	39.7 ± 13.2	54.8 ± 15.8	62.8 ± 14.5	65.3 ± 14.4
Sex, female, *N* (%)	2540 (48.3)	1114 (58.7)	529 (56.2)	504 (60.9)
Race, *N* (%)				
Non-Hispanic White	2478 (70.4)	1112 (75.6)	611 (79.9)	493 (73.1)
Non-Hispanic Black	1057 (10.6)	409 (10.8)	196 (10.7)	212 (15.1)
Hispanic	1356 (13.5)	416 (8.8)	179 (5.5)	144 (5.8)
Other	228 (5.5)	72 (4.7)	28 (4.0)	39 (5.9)
Education, *N* (%)				
Less than high school	1193 (14.3)	614 (19.5)	384 (27.6)	386 (33.1)
High school	1195 (24.4)	513 (27.4)	277 (30.3)	211 (29.3)
Some college/associated education	1560 (32.7)	528 (31.1)	226 (26.4)	204 (27.6)
College graduate or more	1167 (28.6)	352 (22.0)	127 (15.7)	80 (10.0)
Annual household Income (USD), *N* (%)				
0–19,999	802 (11.1)	478 (18.2)	335 (27.3)	385 (39.2)
20,000–44,999	1533 (27.0)	686 (33.0)	354 (38.3)	266 (34.6)
45,000–74,999	1149 (26.2)	391 (25.6)	143 (21.2)	120 (18.4)
≥ 75,000	1336 (35.7)	335 (23.3)	107 (13.2)	55 (7.8)
Marital status, *N* (%)				
Married	3376 (67.8)	1245 (65.4)	569 (59.9)	402 (50.0)
Widowed	129 (1.9)	280 (10.7)	225 (16.8)	260 (24.2)
Divorced or separated	500 (10.2)	294 (14.8)	154 (16.7)	164 (18.7)
Never married	1110 (20.2)	190 (9.1)	65 (6.6)	61 (7.2)
Full-time working, *N* (%)	3819 (80.7)	882 (53.4)	214 (28.1)	72 (11.7)
Smoking status, *N* (%)				
Never	2864 (53.5)	988 (47.4)	411 (40.1)	377 (41.2)
Former	1021 (20.5)	600 (29.7)	414 (38.1)	346 (37.7)
Current	1234 (26.0)	421 (22.9)	189 (21.8)	165 (21.1)

The percentages and mean values are weighted

*USD* United States Dollar

Regarding objective 1 (to evaluate the relationship between individual nutrition-related parameters and frailty), many but not all nutrition-related parameters—especially those related to self-reported intake—varied in relation to the degree of frailty. The proportion of individuals who had abnormal dietary intakes differed significantly between FI groups in almost all variables, except high intake of saturated fat (%), vitamin A, iron, zinc, copper, selenium, and caffeine, and low intake of vitamin A and vitamin C (Table 2). Related to anthropometric measurement, only the percentage of individuals who were underweight and had low subscapular skinfold thickness did not significantly differ between FI groups (Table 3). Similarly, the proportion of individuals who had abnormal blood tests differed significantly between FI groups in almost all variables, except low MCV, low levels of folate in red blood cell and plasma glucose, and high levels of haemoglobin, serum beta-carotene, serum lutein/zeaxanthin, and serum iron (Table 4).

**Table 2:** Number of participants with abnormal range of daily nutrient intakes by frailty level

Nutrients, *N* (%)*		Frailty index score			
		≤ 0.1 *N* = 5119	> 0.1 to 0.2 *N* = 2009	> 0.2 to 0.3 *N* = 1014	> 0.3 *N* = 888
Energy (*N* = 8614)	Low	2218 (44.4)	1157 (55.3)	297 (63.8)	203 (71.7)
Energy per weight (*N* = 8510)	Low	1950 (39.8)	1051 (54.1)	605 (60.9)	566 (69.7)
	High	1479 (30.8)	307 (17.4)	108 (13.9)	64 (7.9)
Protein (*N* = 8614)	Low	821 (15.6)	450 (20.9)	297 (27.5)	303 (33.5)
Protein per weight (*N* = 8510)	Low	1524 (29.0)	955 (46.8)	563 (55.0)	524 (63.6)
Carbohydrate (*N* = 8614)	Low	1068 (22.8)	608 (31.1)	357 (35.5)	360 (41.2)
Simple sugar (*N* = 8614)	High	4633 (94.6)	1778 (92.9)	896 (93.1)	758 (91.7)
Dietary fiber per energy (*N* = 8613)	Low	4590 (94.6)	1713 (91.0)	870 (91.9)	755 (92.8)
Percentage of fat (*N* = 8614)	Low	119 (2.0)	83 (3.6)	41 (4.2)	46 (4.6)
	High	4413 (91.1)	1650 (88.1)	799 (85.0)	670 (82.7)
Percentage of saturated fat (*N* = 8613)	High	2827 (59.6)	1078 (59.0)	554 (57.4)	479 (60.8)
Cholesterol (*N* = 8614)	High	1924 (39.2)	652 (33.4)	312 (30.9)	255 (28.5)
Vitamin A, RAE (*N* = 8614)	Low	3725 (75.0)	1502 (76.8)	745 (76.1)	647 (76.7)
	High	31 (0.7)	5 (0.1)	11 (1.0)	4 (0.5)
Vitamin C (*N* = 8614)	Low	2903 (62.2)	1165 (61.8)	598 (63.2)	516 (65.1)
	High	0 (0.0)	0 (0.0)	0 (0.0)	1 (0.1)
Vitamin E (*N* = 8614)	Low	4548 (92.4)	1814 (93.2)	931 (94.9)	802 (95.9)
Vitamin K (N = 8614)	Low	3754 (74.4)	1503 (76.0)	776 (78.0)	679 (80.6)
Thiamin (*N* = 8614)	Low	1411 (27.3)	700 (34.3)	362 (35.2)	375 (42.6)
Riboflavin (*N* = 8614)	Low	831 (14.5)	359 (15.7)	189 (17.4)	212 (23.6)
Niacin (*N* = 8614)	Low	981 (18.0)	544 (25.3)	301 (26.2)	332 (37.1)
	High	1020 (23.0)	223 (13.1)	95 (13.0)	65 (8.7)
Pyridoxine (*N* = 8614)	Low	1596 (32.2)	898 (43.7)	507 (47.9)	470 (54.0)
Folate (*N* = 8614)	Low	2751 (54.8)	1236 (63.3)	658 (64.6)	606 (71.3)
	High	138 (3.2)	38 (2.1)	19 (2.7)	10 (1.3)
Cobalamin (*N* = 8614)	Low	1252 (24.5)	593 (28.5)	307 (30.5)	287 (32.8)
Calcium (*N* = 8614)	Low	3150 (63.7)	1457 (73.4)	787 (78.4)	698 (81.0)
	High	125 (2.8)	30 (1.8)	9 (1.2)	4 (0.8)
Phosphorous (*N* = 8614)	Low	551 (10.1)	322 (14.7)	187 (18.5)	217 (24.7)
	High	29 (0.5)	8 (0.5)	1 (0.3)	0 (0.0)
Magnesium (*N* = 8614)	Low	3656 (74.2)	1526 (76.9)	828 (82.7)	731 (87.1)
Iron (*N* = 8614)	Low	1750 (34.7)	579 (30.7)	223 (23.3)	228 (29.0)
	High	65 (1.4)	21 (1.1)	7 (1.0)	5 (0.7)
Zinc (*N* = 8614)	Low	1863 (36.3)	898 (42.8)	531 (49.7)	468 (52.5)
	High	56 (1.2)	14 (0.8)	8 (1.0)	3 (0.3)
Copper (*N* = 8614)	Low	1322 (25.5)	663 (31.9)	369 (34.8)	379 (44.4)
	High	10 (0.3)	1 (0.0)	2 (0.1)	1 (0.1)
Sodium (*N* = 8614)	Low	359 (6.2)	183 (8.0)	81 (7.5)	117 (12.4)
	High	3742 (79.2)	1219 (65.8)	599 (64.5)	435 (54.2)
Potassium (*N* = 8614)	Low	4484 (91.4)	1799 (92.4)	935 (95.6)	810 (96.7)
Selenium (*N* = 8614)	Low	571 (10.8)	344 (16.9)	203 (20.4)	228 (26.4)
	High	15 (0.3)	8 (0.5)	1 (0.1)	0 (0.0)
Caffeine (*N* = 8614)	High	489 (14.2)	191 (13.5)	82 (12.3)	80 (11.4)
Alcohol (*N* = 8614)	High	885 (21.7)	270 (16.8)	111 (12.9)	59 (8.8)
Linoleic acid (*N* = 8614)	Low	2414 (47.9)	1030 (51.3)	562 (54.7)	531 (62.1)
α-Linolenic acid (*N* = 8614)	Low	2491 (49.8)	1100 (53.8)	603 (58.4)	552 (63.9)
Fish oil (*N* = 8614)	Low	4343 (88.7)	1700 (88.5)	872 (90.6)	764 (91.1)

*RAE* retinol activity equivalents

*The percentages are weighted

**Table 3:** Number of participants with abnormal range of anthropometric measurement by frailty level

Anthropometric measurements, *N* (%)*		Frailty index score			
		≤ 0.1 *N* = 5119	> 0.1 to 0.2 *N* = 2009	> 0.2 to 0.3 *N* = 1014	> 0.3 *N* = 888
Body mass index (*N* = 8873)	Underweight	91 (1.9)	22 (1.3)	17 (1.8)	10 (1.2)
	Overweight	1816 (34.5)	702 (33.8)	341 (31.5)	244 (29.3)
	Obese	1519 (28.6)	735 (38.9)	408 (44.1)	359 (44.2)
Body weight change in past 1 year (*N* = 8852)	Loss > 10%	381 (6.8)	194 (9.7)	122 (10.9)	151 (15.6)
	Gain > 10%	872 (13.7)	252 (12.1)	115 (13.3)	104 (14.0)
Waist circumference (*N* = 8644)	High	3444 (67.2)	1603 (82.2)	815 (85.9)	643 (86.1)
Triceps skinfold (*N* = 7885)	Low	538 (11.3)	147 (8.1)	84 (8.6)	76 (10.3)
	High	415 (9.3)	184 (12.3)	108 (15.9)	93 (13.5)
Subscapular skinfold (*N* = 6884)	Low	428 (11.1)	143 (9.3)	66 (8.4)	62 (11.2)
	High	281 (7.2)	140 (9.0)	62 (10.0)	45 (6.8)

*The percentages and mean values are weighted

**Table 4:** Number of participants with abnormal range of blood levels by frailty level

Blood tests, *N* (%)*		Frailty index score			
		≤ 0.1 *N* = 5119	> 0.1 to 0.2 *N* = 2009	> 0.2 to 0.3 *N* = 1014	> 0.3 *N* = 888
Total lymphocyte count (*N* = 8965)	Low	862 (17.8)	451 (20.9)	272 (24.2)	304 (34.6)
Haemoglobin (*N* = 9017)	Low	304 (3.4)	224 (7.4)	175 (12.6)	216 (20.9)
	High	40 (1.0)	25 (1.4)	13 (2.1)	9 (0.8)
Mean corpuscular volume (*N* = 9017)	Low	170 (2.4)	130 (5.3)	30 (2.3)	43 (4.2)
	High	43 (0.9)	74 (3.7)	56 (5.8)	56 (6.6)
Albumin (*N* = 8916)	Low	308 (1.8)	84 (1.8)	28 (2.2)	68 (7.2)
Vitamin A (*N* = 8889)	Low	1 (0.0)	2 (0.1)	3 (0.1)	5 (0.6)
	High	168 (4.4)	148 (8.9)	128 (13.7)	159 (19.0)
Vitamin C (*N* = 8886)	Low	264 (6.6)	147 (7.4)	78 (8.3)	82 (8.0)
	High	77 (1.8)	66 (3.4)	44 (4.6)	36 (4.4)
Vitamin D (*N* = 8976)	Low	1906 (29.4)	740 (30.5)	422 (35.6)	438 (44.6)
	High	59 (1.5)	9 (0.6)	2 (0.2)	2 (0.3)
Pyridoxine (*N* = 8926)	Low	869 (15.0)	380 (16.5)	206 (19.4)	231 (25.6)
Folate, RBC (*N* = 8959)	Low	249 (4.1)	73 (2.7)	40 (3.1)	31 (3.5)
Cobalamin (*N* = 8865)	Low	112 (2.0)	50 (2.4)	43 (4.4)	39 (5.3)
α-carotene (*N* = 8885)	Low	1045 (21.4)	396 (20.4)	220 (22.5)	241 (30.3)
	High	562 (11.4)	223 (11.1)	72 (6.1)	54 (6.6)
β-carotene (*N* = 8501)	Low	908 (19.5)	345 (20.0)	197 (21.9)	189 (24.7)
	High	565 (11.9)	277 (13.4)	131 (11.8)	101 (11.0)
β-cryptoxanthin (*N* = 8865)	Low	619 (15.5)	368 (21.4)	247 (28.8)	257 (35.6)
	High	876 (12.3)	294 (12.1)	122 (8.7)	76 (7.0)
Lutein/Zeaxanthin (*N* = 8889)	Low	1131 (26.5)	531 (32.0)	307 (34.8)	346 (46.5)
	High	229 (3.8)	109 (4.8)	46 (4.5)	34 (3.5)
Lycopene (*N* = 8889)	Low	584 (10.7)	401 (16.3)	317 (29.2)	369 (40.3)
	High	666 (14.0)	163 (10.0)	55 (6.3)	34 (5.4)
Iron, serum (*N* = 8910)	Low	669 (11.6)	309 (13.8)	145 (15.3)	180 (20.8)
	High	84 (1.8)	22 (1.1)	10 (1.0)	7 (1.0)
Creatinine (*N* = 8916)	Low	337 (3.4)	103 (3.7)	40 (3.3)	30 (4.1)
	High	68 (1.2)	145 (6.0)	166 (13.9)	232 (24.7)
Total cholesterol (*N* = 8950)	High	2380 (46.1)	1053 (52.6)	445 (44.2)	367 (43.7)
Triglyceride (*N* = 8911)	High	1574 (29.1)	734 (39.2)	402 (42.2)	370 (44.1)
HDL-c (*N* = 8949)	Low	1453 (30.1)	576 (30.9)	290 (29.8)	312 (37.9)
LDL-c (*N* = 4161)	High	789 (32.7)	318 (32.5)	119 (24.0)	115 (29.0)
Glucose (*N* = 8916)	Low	141 (2.0)	25 (1.0)	16 (1.4)	20 (2.6)
	High	814 (15.3)	666 (31.5)	439 (39.7)	423 (46.5)
Homocysteine (*N* = 8979)	High	21 (0.5)	25 (1.1)	26 (2.1)	46 (5.0)

*HDL-c* high-density lipoprotein cholesterol, *LDL-c* low-density lipoprotein cholesterol, *RBC* red blood cell

*The percentages are weighted

**Fig. 1:**
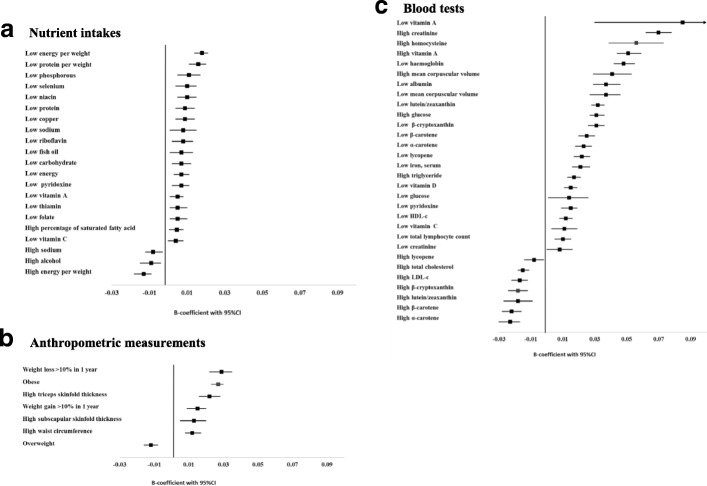
Association between abnormal nutritional-related parameters and frailty. **a** Nutrient intakes. **b** Anthropometric measurements. **c** Blood tests. HDL-c, high-density lipoprotein cholesterol; LDL-c, low-density lipoprotein cholesterol; MCV, mean corpuscular volume. All analyses were adjusted for age, sex, race, energy intake, educational level, marital status, employment status, smoking, and study cohort except for energy, energy per weight, and dietary fiber per energy which were not adjusted for energy intake

Linear regression models, adjusted for the potential covariates, revealed statistically significant associations between frailty and the inappropriate intake of many nutrients (Table 7 in Appendix), the abnormal range of many anthropometric measures (Table 8 in Appendix), and the abnormality of many nutrition-related blood tests (Table 9 in Appendix). To summarize, frailty was associated with 19 nutrient intakes (Fig. 1a). Low energy intake per weight showed the highest positive correlation with frailty (*β*-coefficient 0.018, 95%CI 0.014–0.021) followed by low protein per weight intake (0.016, 0.011–0.020), whereas high consumption of energy per weight, sodium, and alcohol were significantly associated with lower FI score. With regard to anthropometric measurements, only being overweight was significantly associated with lower frailty. Obesity, high waist circumference, triceps and subscapular skinfold thickness, and body weight change (loss and gain more than 10%) were significantly associated with higher FI score (Fig. 1b). Almost all blood tests (21/23) were significantly correlated with frailty. The highest association was found in low serum vitamin A (*β*-coefficient 0.085, 95%CI 0.030–0.139). High serum levels of alpha-carotene, beta-carotene, beta-cryptoxanthin, lutein/zeaxanthin, lycopene, total cholesterol, and LDL-c were inversely associated with FI score (Fig. 1c).

**Fig. 2:**
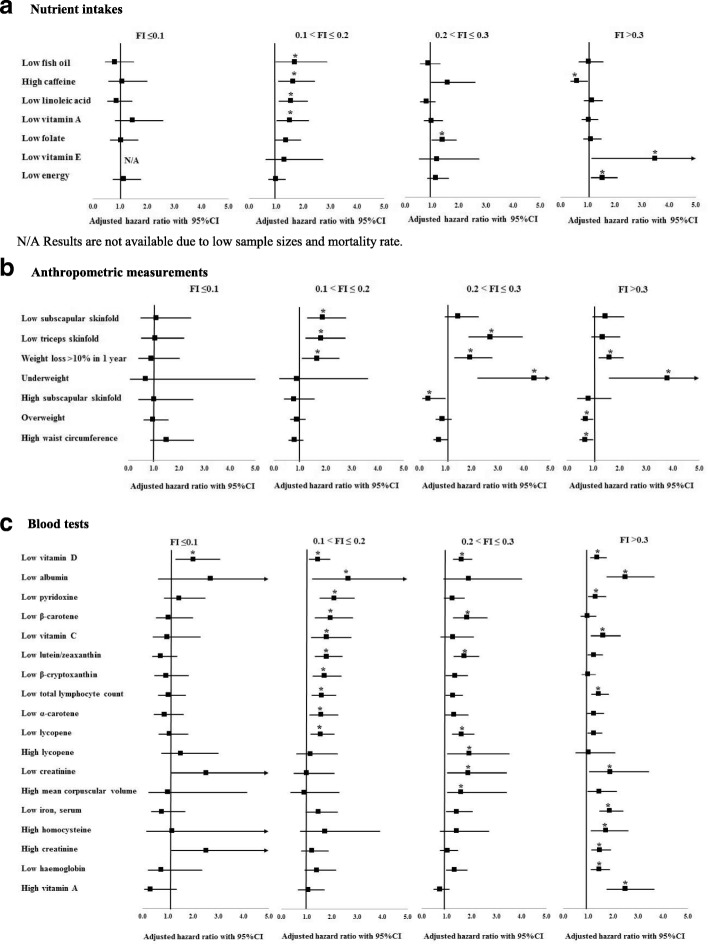
Association between abnormal nutritional-related parameters and mortality across levels of frailty. **a** Nutrient intakes. N/A, results are not available due to low sample sizes and mortality rate. **b** Anthropometric measurements. **c** Blood tests. FI, frailty index. All analyses were adjusted for age, sex, race, energy intake, educational level, marital status, employment status, smoking, and study cohort except for energy and energy per weight which were not adjusted for energy intake. *p value < 0.05

Results related to the relationship of the nutrition-related parameters with mortality risk (objective 2) are presented in Fig. 2 and Tables 10, 11, and 12 in Appendix. To summarize, only one abnormal blood test (low vitamin D which was associated with mortality risk at all grades of frailty) showed a relationship with mortality in people with FI ≤ 0.1; four nutrient intakes, three anthropometric measurements, and ten blood tests in people with 0.1–0.2 FI; one nutrient intake, four anthropometric measurements, and six blood tests in people with 0.2–0.3 FI; and three nutrient intakes, three anthropometric measurements, and ten blood tests in people with FI > 0.3. Participants with FI > 0.1 who reported that they lost more than 10% of their weight in the past year had higher mortality risk. Being underweight and low serum creatinine levels were associated with higher mortality risk in individuals with FI > 0.2. Being overweight, having high waist circumference, and caffeine consumption were significantly associated with lower mortality risk in individuals with FI > 0.3.

**Fig. 3:**
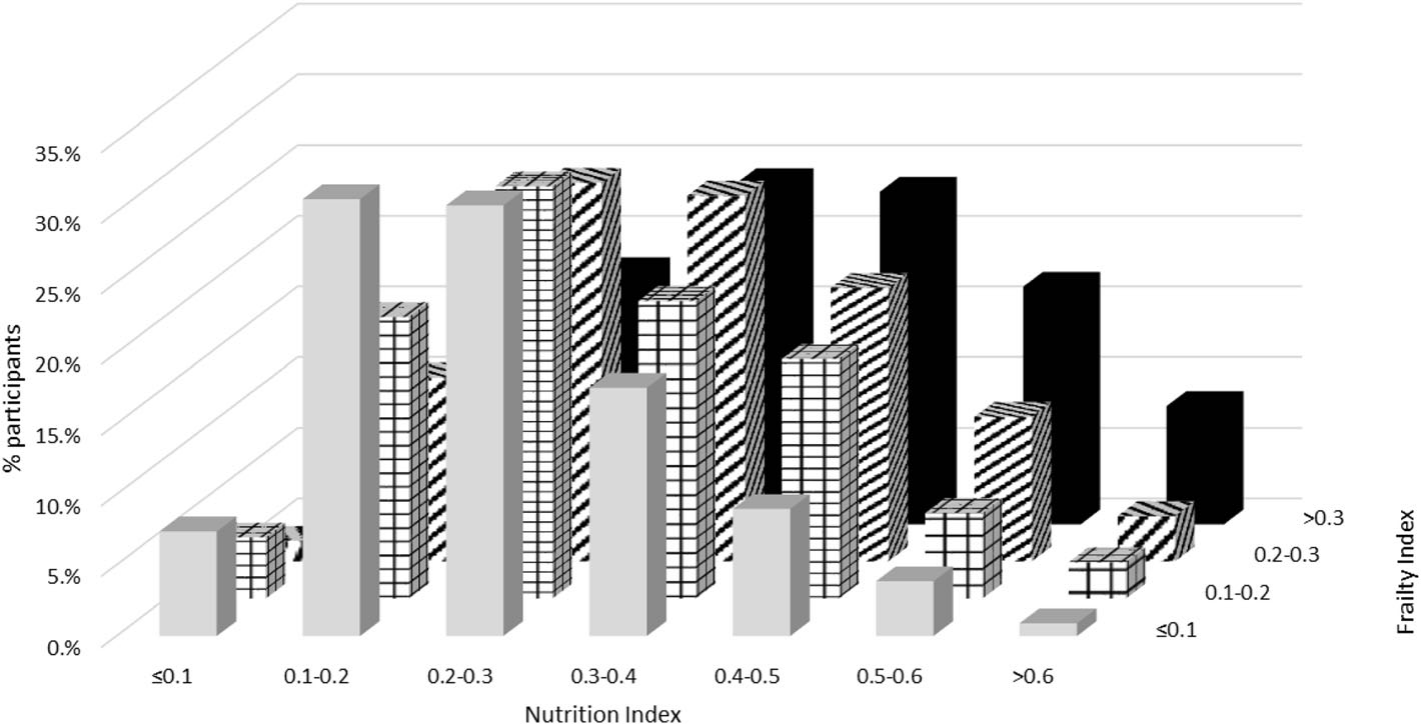
Percentage of participants in each level of nutritional index score by frailty level. The percentages are weighted

**Fig. 4:**
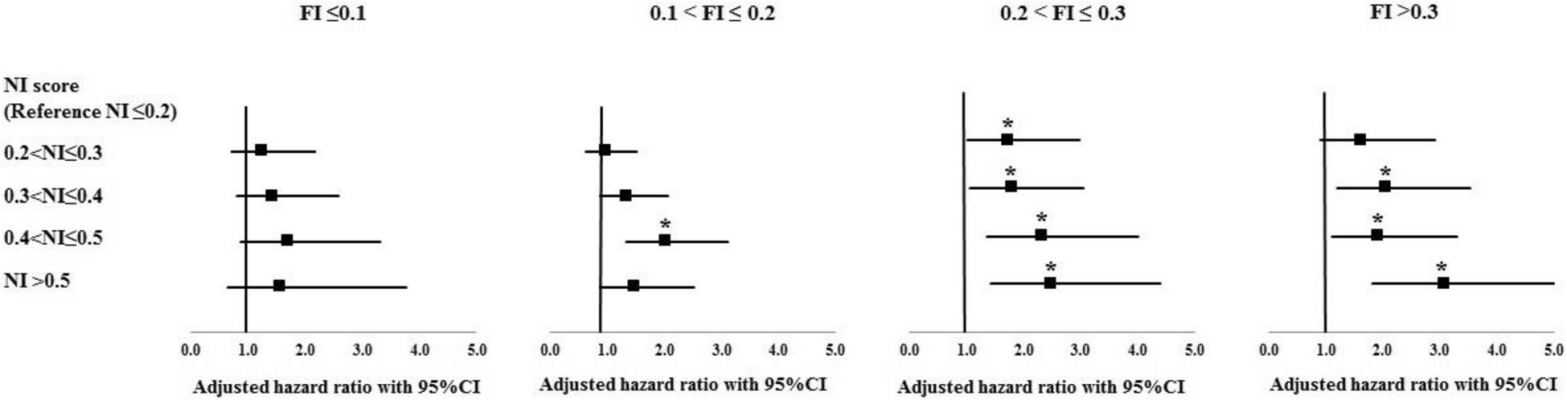
Association between nutritional index and mortality across levels of frailty. FI, frailty index; NI, nutritional index. All analyses were adjusted for age, sex, race, educational level, marital status, employment status, smoking, and study cohort except for energy and energy per weight which were not adjusted for energy intake. *p value < 0.05

**Fig. 5:**
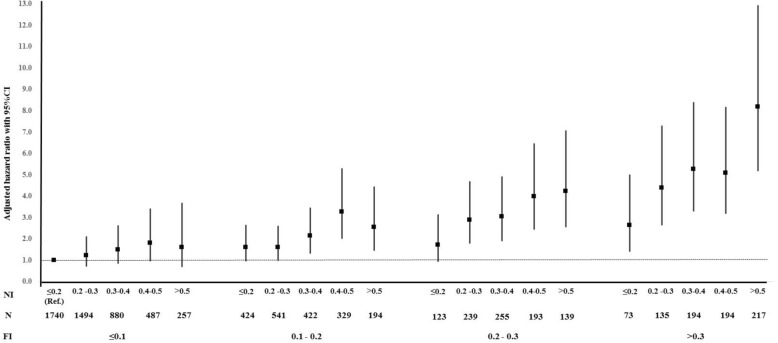
Combined effect of frailty and nutrition on mortality. FI, frailty index; NI, nutritional index. All analyses were adjusted for age, sex, race, educational level, marital status, employment status, smoking, and study cohort

Regarding objective 3 (to examine whether combining nutrition-related parameters in an index predicts mortality risk across frailty levels), we could not calculate the NI score for 500 individuals due to missing > 20% of the nutritional parameters included in the index (total included *n* = 8530). Overall, 393 (5.8%) of the participants had an NI score less than 0.1 (abnormality in ≤ 4 of the 41 parameters examined). This proportion decreased with higher frailty, from 7.4% among those with FI < 0.1 to 0.7% among those with FI > 0.3 (Fig. 3 and Table 13 in Appendix). The weighted mean NI score was 0.29 ± 0.13 (range 0.00–0.79) and was significantly higher for those people with higher frailty levels: 0.26 ± 0.12 for FI ≤ 1, 0.31 ± 0.13 for 0.1–0.2 FI, 0.35 ± 0.13 for 0.2–0.3 FI, and 0.40 ± 0.14 for FI > 0.3. Higher NI score was significantly associated with higher frailty (*β*-coefficient 1.46, 95%CI 1.459–1.461) and higher mortality risk (HR per 0.1 NI score 1.30, 95%CI 1.23–1.36) after adjusting the models for potential covariates. After adjusting the survival analysis additionally for the FI, the HR per 0.1 NI score was 1.19 (95%CI 1.13–1.26). When analysis was stratified by frailty level, higher NI scores were significantly correlated with higher mortality in individual with FI > 0.1; HR per 0.1 NI score was 1.17 (1.06–1.30) for those with 0.1–0.2 FI, 1.20 (1.08–1.32) for those with 0.2–0.3 FI, and 1.27 (1.16–1.38) for those with FI > 0.3 (Fig. 4 and Table 14 in Appendix). When we examined the joint effect of nutrition and frailty status on mortality, we found a dose-response relationship (Fig. 5 and Table 15 in Appendix). People with FI > 0.3 had a higher mortality risk regardless of nutrition status, whereas having an FI ≤ 0.1 was not associated with frailty even for those with NI > 0.5. People with FI > 0.3 and NI > 0.5 had the highest mortality risk (HR 8.17, 95%CI 5.16–12.94).

## Discussion

This observational study aimed to improve our understanding of the relationship between frailty and nutrition. As expected, we found that the two are related. When we looked at one nutritional parameter at a time (objective 1), the details are complicated: most but not all of the abnormal nutrition-related parameters included in NHANES were related to frailty (19/34 of nutrient intakes, all 5 anthropometric measurements and 21/23 of blood tests). Nevertheless, fewer than half were individually associated with higher mortality risk across frailty levels and their impact differed across levels of frailty (objective 2). A relationship with all-cause mortality was found with one parameter in the FI ≤ 0.1 group, 17 parameters in the 0.1–0.2 FI group, 11 parameters in the 0.2–0.3 FI group, and 16 parameters in the > 0.3 FI group. Only low serum vitamin D significantly increased the mortality risk across all levels of frailty. Even so, when we combined the nutrition-related parameters, including those not significantly associated with mortality, the resulting NI strongly predicted mortality risk, especially among those with higher FI scores (objective 3). In short, overall, the results show that frailty and nutrition are related, and for the most part, unless people are in good health, poor nutritional status increases mortality in a dose-dependent fashion, independent of age, sex, marital status, and education.

Several features of these results require additional comment. Regarding the individual items, vitamin D plays an important role in both bone metabolism and non-bony tissue function including skeletal muscles which relate with function in elderly people [58]. Previous observational studies [59, 60] including one using the NHANES III data [61] showed that serum vitamin D levels were correlated with frailty and all-cause mortality in older adults. Moreover, a meta-analysis of RCTs [62] reported the benefit of daily vitamin D supplementation on muscle strength and balance in older people. Concerning cognitive function, severe vitamin D deficiency was also correlated with visual memory decline [63]. The current study confirmed the association between low serum vitamin D levels and both frailty levels and mortality risk across levels of frailty, not only in older people but also in younger people.

According to World Health Organization (WHO), the normal range of weight in healthy adults is defined by body mass index (BMI) or Quetelet index between 18.5 and 24.9 kg/m^2^ [64]. Even so, human physiology and mortality risk factors change with ageing. A previous meta-analysis [65] showed that a BMI < 23 kg/m^2^ was associated with higher mortality risk in older people. BMI alone may not be a good indicator of adiposity in this population and this has been widely demonstrated based on the obesity paradox seen in the older people [66, 67]. The present study showed that obesity was associated with higher frailty but had no relationship with mortality. In contrast, being underweight increased mortality risk in individuals with FI > 0.2 and the mortality risk was lower in people with FI > 0.3 who were overweight. It is possible that body composition and weight change may be better predictors in older people than BMI. This study revealed that excessive fat accumulation, high triceps and subscapular skinfold thickness, waist circumference, and change of body weight (loss and gain) more than 10% in the past year were correlated with higher frailty. Moreover, low triceps skinfold in people with 0.1–0.3 FI and weight loss more than 10% in the past year in people with FI > 0.1 were associated with higher mortality risk.

On the subject of phytochemicals, previous studies [68, 69] showed that low serum carotenoids levels were associated with higher frailty. This study also confirmed that low serum alpha-carotene, beta-carotene, beta-cryptoxanthin, lutein/zeaxanthin, and lycopene levels increased the risks of frailty and mortality; high serum levels of these carotenoids were associated with lower frailty levels. The relationship between the amount of dietary carotenoid intakes and their serum levels in older adults should be explored further. Recommending carotenoids-rich fruits and vegetables consumption could be the focus of dietary interventions to improve frailty status.

This study illustrates the virtue of considering deficit accumulation as a means of providing context in age-related disorders. As put pithily in a 2014 Nature commentary, “the problems of old age come as a package” [70]. Deficit accumulation indices can quantify those packages of age-associated problems [71] and have been used by our group and others in a variety of contexts to quantify the cumulative impact of brain MRI changes [72], social vulnerability measures [73], laboratory measures [74], and ageing biomarkers [75]. An NI, constructed using the deficit accumulation approach, was a stronger prediction of frailty and mortality risk than were single nutritional parameters. This study, similarly to previous studies [76, 77], highlights that the accumulation of small deficits, even those that may not result in clinically detectable problems, corresponds to the ability of the organism to respond and recover from stressors [78]. A recent report noted the benefit to considering 11 nutrition-related parameters in mortality prediction, but did not evaluate frailty [40]. The findings from that work do not contradict our key clinical message: patient management should reflect not just nutritional parameters that cross an illness threshold, but the overall nutritional status.

In addition, there appears to be some merit in broader modeling of the nutrition risk as part of age-related deficit accumulation [79]. For example, the doubling time of biomarker deficits appears to be longer than laboratory ones, which in turn are longer than clinical deficits [74, 75, 80], something which appears to reflect their relative connectivity as nodes in a network. How the various types of nutritional deficits fit in this spectrum is of interest, with an initial hypothesis that their variable relationships with mortality might reflect their connectivity (or other network properties). Recent work suggests that information theory might help better analyse factors that influence the health trajectories of individuals [79], offering pragmatic new approaches to studying age-related disease [81].

Here, participants with low energy consumption for their body weight were more likely to be frail. Lower than recommended calorie intake can cause malnutrition; high levels of frailty are common among malnourished people [8]. We also showed a strong association between frailty and body weight changes of more than 10%, both losing and gaining weight in 1 year. Weight loss is a major sign of malnutrition, is included in most of the nutritional screening tools, and is one of the five criteria used in defining the “frailty phenotype” [82]. Weight loss can be caused not only by loss of fat but also by loss of muscle and bony mass [83]. On the other hand, weight gain leads to more fat mass than muscle mass in sedentary young individuals. The fat accumulation itself is associated with many health deficits, especially the metabolic syndrome and metabolic-related diseases. Even so, how the metabolic syndrome and frailty interact in relation to mortality appears to change across the life course [84].

The causes of frailty may be different at each age group. For example, younger people may accumulate deficits due to a chronic condition whereas older people may accumulate deficits even when few comorbidities are present [85]. Similarly, nutritional problems are altered across the lifespan. For example, older people may require more protein and calcium intake than do younger people [45, 86] whereas the requirement for iron typically declines after the menopause [52]. Here, we recognized this by using cutoff points of normal intake according to the recommendation for each age and gender group. Even so, the effect of abnormal nutrition on frailty can be different in each age group and future interventional studies need to investigate this.

We used publicly available data from NHANES, a large population-based study with a well-controlled and rigorous protocol. We analysed a huge number of nutrition-related parameters. Mortality was extracted from death certificate data and was examined 5–8 years after testing. However, our data must be interpreted with caution: (a) Due to the cross-sectional design, the causal relationship between frailty and nutrition cannot be examined and the duration of exposure to each parameter cannot be explored. For example, here, daily alcohol consumption of more than 2 standard drinks (28 g) in men and 1 standard drink in women (14 g) was associated with lower frailty but was not related with mortality risk. Nevertheless, alcohol consumption more than 3 standard drinks (42 g) per day was not associated with frailty (data not shown). (b) Since dietary data (including alcohol use) were recorded by 24-h recall, day-to-day variation could not be counted, and food intake could be altered along the study period. (c) People who have chronic abnormal serum levels of some nutrients may have experienced temporally normal levels during testing.

The absence of longitudinal data also makes it difficult to discern age from period and cohort effects. Our data do however demonstrate that both frailty and nutritional deficiencies can be detected at all adult ages. Nutritional deficiencies, at least in the aggregate, can also be seen more commonly at higher ages and with frailty, and increase the lethality of frailty. Here, for similar levels of deficit accumulation, at all ages, impaired nutrition reduced survival in people whose FI score were higher than 0.1.

## Conclusions

This study revealed that most nutritional parameters were related with frailty, but the impact of individual parameters on mortality differed across levels of frailty. Only low vitamin D was associated with higher levels of frailty and higher risk for mortality across all levels of frailty. Weight loss more than 10% in the past year also increased mortality risk, except in very fit people. Nevertheless, mortality risk was decreased by being overweight, having high waist circumference and subscapular skinfold and consuming more than 400 mg of caffeine daily in people FI > 0.3. Even though many nutrition-related parameters were not significantly associated with mortality, we found that in people with FI > 0.1, they strongly predicted mortality risk when combined in an index. The combined effect of frailty and nutrition deficits had the most impact on mortality risk. Balanced nutritional interventions appear to be reasonable approaches to remediating frailty. Further studies are needed to examine the impact of nutritional interventional studies on frailty levels and to evaluate whether the number of nutritional deficits relates to other health outcomes such as hospitalization, institutionalization, and quality of life.

## Appendix

**Table 5:** Normal range of parameter

Parameter	Normal range	Score in nutritional index	
		0	1
Nutrient intakes			
Energy (kcal/day)	M ≥ 2400, F ≥ 1800	Normal range	M < 2400, F < 1800
Energy per weight (kcal/kg/day)	25–35	≥ 25	< 25
Protein (g/day)	M ≥ 56, F ≥ 46	Normal range	M < 56, F < 46
Protein per weight (g/kg/day)	< 65 years, ≥ 0.8 ≥65 years, ≥ 1	Normal range	< 65 years, < 0.8 ≥ 65 years, < 1
Carbohydrate (g/day)	≥ 180	Normal range	< 180
Simple sugar (mg/day)	M < 36, F < 25	–	–
Dietary fiber (g/1000 kcal/day)	> 14	–	–
Percentage of fat (%)	20–35	–	–
Percentage of saturated fat (%)	< 10	–	–
Cholesterol (mg/day)	< 300	–	–
Vitamin A, RAE (mcg/day)	M 900–3000, F 700–3000	–	–
Vitamin C (mg/day)	M 90–2000, F 75–2000	–	–
Vitamin E (mg/day)	15–1000	–	–
Vitamin K (mcg/day)	M ≥ 120, F ≥ 90	Normal range	M < 120, F < 90
Thiamin (mg/day)	M ≥ 1.2, F ≥ 1.1	Normal range	M < 1.2, F < 1.1
Riboflavin (mg/day)	M ≥ 1.3, F ≥ 1.1	Normal range	M < 1.3, F < 1.1
Niacin (mg/day)	M 16–35, F 14–35	–	–
Pyridoxine (mg/day)	≤ 50 years, 1.3–100 > 50 years, M 1.7–100 > 50 years, F 1.5–100	Normal range	≤50 years, < 1.3 or > 100 > 50 years, M < 1.7 or > 100 > 50 years, F < 1.5 or > 100
Folate (mcg/day)	400–1000	Normal range	< 400 or > 1000
Cobalamin (mcg/day)	≥ 2.4	Normal range	< 2.4
Calcium (mg/day)	M ≤ 70 years, 1000–2500 M > 70 years, 1200–2500 F ≤ 50 years, 1000–2500 F > 50 years, 1200–2500	Normal range	M ≤ 70 years, < 1000 or > 2500 M > 70 years, < 1200 or > 2500 F ≤ 50 years, < 1000 or > 2500 F > 50 years, < 1200 or > 2500
Phosphorous (mg/day)	700–4000	Normal range	< 700 or > 4000
Magnesium (mg/day)	M ≥ 420, F ≥ 320	Normal range	M < 420, F < 320
Iron (mg/day)	M 8–45 F ≤ 50 years, 18–45 F > 50 years, 8–45	–	–
Zinc (mg/day)	M 11–40, F 8–40	Normal range	M < 11 or > 40, F < 8 or > 40
Copper (mg/day)	0.9–10	Normal range	< 0.9 or > 10
Sodium (mg/day)	≤ 50 years, 1500–2300 > 50–70 years, 1300–2300 > 70 years, 1200–2300	–	–
Potassium (mg/day)	≥ 4700	–	–
Selenium (mcg/day)	55–400	Normal range	< 55 or > 400
Caffeine (mg/day)	≤ 400	–	–
Alcohol (g/day)	M ≤ 28, F ≤ 14	M > 28, F > 14	M ≤ 28, F ≤ 14
Linoleic acid (g/day)	≤ 50 years, M ≥ 17, F ≥ 12 > 50 years, M ≥ 14, F ≥ 11	Normal range	≤ 50 years, M < 17, F < 12, > 50 years, M < 14, F < 11
α-Linolenic acid (g/day)	M ≥ 1.6, F ≥ 1.1	Normal range	M < 1.6, F < 1.1
Fish oil (g/day)*	≥ 0.25	–	–
Anthropometric measurements			
Body mass index (kg/m^2^)	18.5–24.9**	18.5–29.9	< 18.5 or ≥ 30
Body weight change in past 1 year (%)	≤ 10	–	–
Waist circumference (cm)	M < 94, F < 80	Normal range	M > 94, F > 80
Triceps skinfold (mm)	M 7.5–24.3, F 14–33.7	–	–
Subscapular skinfold (mm)	M10.3–30.5, F10.3–33.9	–	–
Blood tests			
Total lymphocyte count (cells/mm^3^)	> 1500	Normal range	≤ 1500
Haemoglobin (g/dL)	M 13.5–18, F 12–16	Normal range	M < 13.5 or > 18, F < 12 or > 16
MCV (fL)	80–100	–	–
Albumin (g/L)	35–55	–	–
Vitamin A (mcmol/L)	0.35–3.00	Normal range	< 0.35 or > 3.00
Vitamin C (mg/dL)	0.2–2.0	Normal range	< 0.2 or > 2.0
Vitamin D (ng/mL)	20–50	–	–
Pyridoxine (nmol/L)	> 20	–	–
Folate, RBC (ng/mL)	≥ 140	–	–
Cobalamin (pg/L)	> 200	Normal range	≤ 200
α-carotene (mcg/dL)	1.3–9.2	–	–
β-carotene (mcg/dL)	6.4–35.1	–	–
β-cryptoxanthin (mcg/dL)	4.0–16.4	≥ 4.0	< 4.0
Lutein/Zeaxanthin (mcg/dL)	11.1–33.0	–	–
Lycopene (mcg/dL)	11.9–36.1	≥ 11.9	< 11.9
Iron, serum (mcg/dL)	50–180	–	–
Creatinine (mg/dL)	M 0.80–1.40, F 0.56–1.00	Normal range	M < 0.80 or > 1.40, F < 0.56 or > 1.00
Total cholesterol (mg/dL)	< 200	–	–
Triglyceride (mg/dL)	< 150	Normal range	≥ 150
HDL-c (mg/dL)	M > 40, F > 50	–	–
LDL-c (mg/dL)	< 130	–	–
Glucose (mg/dL)	70–100	Normal range	< 70 or > 100
Homocysteine (mcmol/L)	≤ 21.6	Normal range	> 21.6

*F* female, *HDL-c* high-density lipoprotein cholesterol, *LDL-c* low-density lipoprotein cholesterol, *M* male, *MCV* mean corpuscular volume, *RAE* retinol activity equivalents, *RBC* red blood cell. These variables were excluded from the nutrition index due to high missing data or no relationship with high frailty. *Dietary fish oil is the combination between docosahexaenoic acid (DHA) and eicosapentaenoic acid (EPA) in dietary intake. ** < 18.5 kg/m^2^ (underweight), 25–29.9 kg/m^2^ (overweight), ≥ 30 kg/m^2^ (obese)

**Table 6:** 36-item frailty index

*Self-reported items*	
1. Angina/angina pectoris	14. Difficulty lifting or carrying
2. Heart attack	15. Difficulty walking between rooms on same floor
3. Coronary heart disease	16. Difficulty standing up from armless chair
4. Stroke	17. Difficulty getting in and out of bed
5. Thyroid condition	18. Difficulty dressing yourself difficulty
6. Cancer	19. Difficulty grasping/holding small objects
7. Arthritis	20. Difficulty attending social event
8. High blood pressure	21. Self-reported health
9. Diabetes mellitus	22. Frequency of healthcare use
10. Weak/failing kidneys	23. Health compared to 1 year ago
11. Confusion or inability to remember things	24. Overnight hospital stays
12. Difficulty managing money	25. Medications
13. Difficulty stooping, crouching, kneeling	
Laboratory items	
26. Pulse rate (60–99 bpm)	32. Red cell distribution width (≤ 14.6%)
27. Systolic blood pressure (90–140 mmHg)	33. Lactate dehydrogenase (≤ 190 U/L)
28. Pulse pressure (30–60 mmHg)	34. Alkaline phosphatase (≤ 115 U/L)
29. Platelet count SI (150–450 unit 1000 cells/uL)	35. Uric acid (M: 240–510, F: 160–430 umol/L)
30. Blood urea nitrogen (3–20 mg/dL)	36. Total calcium (2.0–2.5 mmol/L)
31. Bicarbonate (≤ 28 mmol/L)	

*F* female, *M* male

**Table 7:** Association between abnormal nutrient intakes and frailty

Nutrients		Linear regression analysis	
		*β*-coefficient (95%CI)	*p* value
Energy	Low	0.007 (0.003, 0.011)	0.001*
Energy per weight	Low	0.018 (0.014, 0.021)	< 0.001*
	High	− 0.013 (− 0.018,− 0.009)	< 0.001*
Protein	Low	0.009 (0.004, 0.014)	0.001*
Protein per weight	Low	0.016 (0.011, 0.020)	< 0.001*
Carbohydrate	Low	0.007 (0.002, 0.012)	0.004*
Simple sugar	High	− 0.004 (− 0.012, 0.003)	0.267
Dietary fiber per energy	Low	0.005 (− 0.002, 0.012)	0.170
Percentage of fat	Low	0.003 (− 0.008, 0.014)	0.597
	High	− 0.001 (− 0.007, 0.005)	0.737
Percentage of saturated fat	High	0.005 (0.001, 0.008)	0.018*
Cholesterol	High	0.003 (− 0.002, 0.007)	0.213
Vitamin A, RAE	Low	0.005 (0.001, 0.010)	0.027*
	High	− 0.018 (− 0.043, 0.006)	0.148
Vitamin C	Low	0.004 (0.001, 0.008)	0.027*
	High	–	
Vitamin E	Low	− 0.004 (− 0.013, 0.004)	0.297
Vitamin K	Low	0.002 (− 0.002, 0.007)	0.328
Thiamin	Low	0.005 (0.001, 0.010)	0.027*
Riboflavin	Low	0.008 (0.002, 0.013)	0.006*
Niacin	Low	0.010 (0.005, 0.015)	< 0.001*
	High	0.000 (− 0.006, 0.006)	0.956
Pyridoxine	Low	0.007 (0.002, 0.011)	0.003*
Folate	Low	0.005 (0.001, 0.010)	0.023*
	High	0.006 (− 0.006, 0.019)	0.339
Cobalamin	Low	0.002 (− 0.002, 0.007)	0.354
Calcium	Low	− 0.003 (− 0.008, 0.002)	0.189
	High	0.011 (− 0.003, 0.025)	0.134
Phosphorous	Low	0.011 (0.005, 0.017)	< 0.001*
	High	0.019 (− 0.010, 0.048)	0.201
Magnesium	Low	0.004 (− 0.002, 0.009)	0.187
Iron	Low	0.001 (− 0.004, 0.006)	0.826
	High	0.012 (− 0.006, 0.030)	0.183
Zinc	Low	0.002 (− 0.003, 0.006)	0.499
	High	0.010 (− 0.010, 0.030)	0.323
Copper	Low	0.009 (0.004, 0.014)	< 0.001*
	High	− 0.014 (− 0.061, 0.032)	0.547
Sodium	Low	0.008 (0.001, 0.015)	0.022*
	High	− 0.008 (− 0.012, − 0.003)	0.002*
Potassium	Low	0.000 (− 0.008, 0.009)	0.971
Selenium	Low	0.010 (0.004, 0.015)	0.001*
	High	0.004 (− 0.032, 0.041)	0.809
Caffeine	High	0.000 (− 0.007, 0.006)	0.911
Alcohol	High	− 0.009 (− 0.015, − 0.004)	0.001*
Linoleic acid	Low	0.004 (0.000, 0.009)	0.060
α-Linolenic acid	Low	0.004 (− 0.001, 0.008)	0.107
Fish oil	Low	0.007 (0.001, 0.013)	0.025*

*RAE* retinol activity equivalents

All analyses were adjusted for age, sex, race, energy intake, educational level, marital status, employment status, smoking and study cohort except for energy, energy per weight and dietary fiber per energy which were not adjusted for energy intake

– Results are not available due to low sample sizes and mortality rate, **p* value < 0.05

**Table 8:** Association between abnormal anthropometric measurements and frailty

Anthropometric measurements		Linear regression analysis	
		*β*-coefficient (95%CI)	*p* value
Body mass index	Underweight	− 0.008 (− 0.023, 0.007)	0.323
	Overweight	− 0.012 (− 0.016, − 0.008)	< 0.001*
	Obese	0.027 (0.023, 0.030)	< 0.001*
Body weight change in past 1 year	Loss > 10%	0.029 (0.022, 0.035)	< 0.001*
	Gain > 10%	0.015 (0.009, 0.020)	< 0.001*
Waist circumference	High	0.012 (0.008, 0.017)	< 0.001*
Triceps skinfold	Low	0.000 (− 0.006, 0.006)	0.989
	High	0.022 (0.016, 0.028)	< 0.001*
Subscapular skinfold	Low	− 0.004 (− 0.011, 0.003)	0.224
	High	0.013 (0.005, 0.020)	0.001*

All analyses were adjusted for age, sex, race, energy intake, educational level, marital status, employment status, smoking and study cohort, **p* value < 0.05

**Table 9:** Association between abnormal blood tests and frailty

Blood tests		Linear regression analysis	
		*β*-coefficient (95%CI)	*p* value
Total lymphocyte count	Low	0.010 (0.005, 0.015)	< 0.001*
Haemoglobin	Low	0.048 (0.042, 0.055)	< 0.001*
	High	− 0.003 (− 0.022, 0.017)	0.798
Mean corpuscular volume	Low	0.037 (0.027, 0.046)	< 0.001*
	High	0.041 (0.029, 0.053)	< 0.001*
Albumin	Low	0.037 (0.029, 0.046)	< 0.001*
Vitamin A	Low	0.085 (0.030, 0.139)	0.002*
	High	0.051 (0.044, 0.059)	< 0.001*
Vitamin C	Low	0.011 (0.003, 0.019)	0.005*
	High	− 0.001 (− 0.013, 0.011)	0.845
Vitamin D	Low	0.015 (0.011, 0.019)	< 0.001*
	High	− 0.150 (− 0.036, 0.006)	0.160
Pyridoxine	Low	0.015 (0.010, 0.020)	< 0.001*
Folate, RBC	Low	− 0.008 (− 0.017, 0.001)	0.093
Cobalamin	Low	0.006 (− 0.005, 0.018)	0.287
α-carotene	Low	0.023 (0.018, 0.028)	< 0.001*
	High	− 0.023 (− 0.030, − 0.017)	< 0.001*
β-carotene	Low	0.025 (0.020, 0.030)	< 0.001*
	High	− 0.022 (− 0.028, − 0.016)	< 0.001*
β-cryptoxanthin	Low	0.031 (0.026, 0.036)	< 0.001*
	High	− 0.017 (− 0.022, − 0.012)	< 0.001*
Lutein/Zeaxanthin	Low	0.032 (0.028, 0.036)	< 0.001*
	High	− 0.018 (− 0.027, − 0.009)	< 0.001*
Lycopene	Low	0.022 (0.017, 0.027)	< 0.001*
	High	− 0.008 (− 0.014, − 0.002)	0.014*
Iron, serum	Low	0.021 (0.016, 0.027)	< 0.001*
	High	0.001 (− 0.015, 0.016)	0.947
Creatinine	Low	0.008 (0.000, 0.016)	0.048*
	High	0.070 (0.062, 0.078)	< 0.001*
Total cholesterol	High	− 0.015 (− 0.019, − 0.011)	< 0.001*
Triglyceride	High	0.017 (0.013, 0.021)	< 0.001*
HDL-c	Low	0.012 (0.008, 0.016)	< 0.001*
LDL-c	High	− 0.018 (− 0.024, − 0.012)	< 0.001*
Glucose	Low	0.014 (0.001, 0.026)	0.029*
	High	0.031 (0.027, 0.036)	< 0.001*
Homocysteine	High	0.056 (0.039, 0.073)	< 0.001*

All analyses were adjusted for age, sex, race, energy intake, educational level, marital status, employment status, smoking and study cohort

*HDL-c* high-density lipoprotein cholesterol, *LDL-c* low-density lipoprotein cholesterol, *RBC* red blood cell, **p* value < 0.05

**Table 10:** Associations between abnormal nutrient intakes and mortality across levels of frailty

Nutrients		Frailty index score							
		≤0.1	*p* value	> 0.1 to 0.2	*p* value	> 0.2 to 0.3	*p* value	> 0.3	*p* value
		HR (95%CI)		HR (95%CI)		HR (95%CI)		HR (95%CI)	
Energy	Low	1.14 (0.74, 1.77)	0.554	1.00 (0.73, 1.35)	0.976	1.16 (0.83, 1.61)	0.384	1.55 (1.14, 2.10)	0.005*
Energy per weight	Low	1.16 (0.72, 1.86)	0.547	0.87 (0.64, 1.18)	0.373	0.93 (0.69, 1.25)	0.632	1.36 (1.00, 1.86)	0.052
	High	0.77 (0.40, 1.47)	0.427	1.11 (0.73, 1.68)	0.620	1.04 (0.63, 1.73)	0.868	1.38 (0.85, 2.25)	0.195
Protein	Low	1.09 (0.64, 1.84)	0.758	0.93 (0.65, 1.32)	0.675	0.84 (0.61, 1.15)	0.266	0.93 (0.71, 1.22)	0.607
Protein per weight	Low	0.90 (0.56, 1.45)	0.670	1.05 (0.76, 1.45)	0.765	0.80 (0.59, 1.09)	0.161	0.84 (0.62, 1.12)	0.238
Carbohydrate	Low	1.25 (0.75, 2.11)	0.394	1.30 (0.92, 1.83)	0.134	1.25 (0.90, 1.74)	0.178	0.88 (0.66, 1.71)	0.367
Simple sugar	High	1.02 (0.46, 2.26)	0.964	0.73 (0.45, 1.17)	0.189	0.92 (0.57, 1.48)	0.720	1.15 (0.77, 1.81)	0.505
Dietary fiber per energy	Low	0.57 (0.32, 1.03)	0.064	1.42 (0.98, 2.26)	0.140	0.99 (0.66, 1.48)	0.941	0.89 (0.62, 1.27)	0.510
Percentage of fat	Low	1.16 (0.31, 4.36)	0.822	0.62 (0.27, 1.43)	0.265	1.42 (0.91, 2.21)	0.335	0.56 (0.31, 1.02)	0.990
	High	1.29 (0.61, 2.73)	0.509	0.68 (0.44, 1.04)	0.072	0.96 (0.76, 122)	0.121	1.049 (0.75, 1.48)	0.784
Percentage of saturated fat	High	0.87 (0.58, 1.32)	0.523	1.27 (0.96, 1.67)	0.092	1.11 (0.85, 1.44)	0.450	0.97 (0.78, 1.21)	0.800
Cholesterol	High	1.17 (0.74, 1.84)	0.502	1.03 (0.76, 1.39)	0.857	1.21 (0.89, 1.66)	0.225	1.08 (0.84, 1.40)	0.552
Vitamin A, RAE	Low	1.47 (0.83, 2.59)	0.184	1.51 (1.03, 2.21)	0.033*	0.99 (0.71, 1.38)	0.935	1.03 (0.78, 1.37)	0.818
	High	1.22 (0.16, 9.41)	0.849	–		2.01 (0.72, 5.63)	0.182	–	
Vitamin C	Low	0.95 (0.62, 1.45)	0.814	1.26 (0.94, 1.67)	0.121	1.19 (0.90, 1.57)	0.223	1.23 (0.97, 1.56)	0.091
	High	–		–		–		–	
Vitamin E	Low	**–**		1.31 (0.62, 2.73)	0.478	1.20 (0.53, 2.74)	0.658	3.49 (1.15, 11.00)	0.033*
Vitamin K	Low	1.13 (0.65, 1.97)	0.657	1.35 (0.92, 1.98)	0.121	1.06 (0.74, 1.51)	0.755	0.97 (0.72, 1.31)	0.842
Thiamin	Low	1.61 (0.99, 2.60)	0.055	1.31 (0.95, 1.80)	0.095	1.17 (0.86, 1.58)	0.310	1.15 (0.89, 1.48)	0.288
Riboflavin	Low	0.83 (0.45, 1.53)	0.548	0.93 (0.62, 1.40)	0.733	1.17 (0.83, 1.65)	0.361	1.00 (0.75, 1.32)	0.992
Niacin	Low	1.15 (0.70, 1.91)	0.581	1.16 (0.83, 1.61)	0.388	1.05 (0.77, 1.43)	0.754	0.99 (0.76, 1.29)	0.945
	High	0.81 (0.39, 1.70)	0.580	1.10 (0.68, 1.77)	0.699	0.58 (0.32, 1.05)	0.074	1.37 (0.89, 2.11)	0.148
Pyridoxine	Low	0.87 (0.55, 1.39)	0.566	0.96 (0.71, 1.31)	0.815	1.26 (0.95, 1.67)	0.106	1.03 (0.80, 1.32)	0.818
Folate	Low	1.04 (0.64, 1.66)	0.885	1.37 (0.98, 1.91)	0.068	1.39 (1.01, 1.91)	0.042*	1.13 (0.84, 1.51)	0.413
	High	1.97 (0.59, 6.57)	0.269	1.35 (0.53, 3.45)	0.526	1.00 (0.31, 3.21)	0.996	0.77 (0.19, 3.16)	0.714
Cobalamin	Low	1.10 (0.68, 1.77)	0.695	1.09 (0.79, 1.49)	0.605	1.15 (0.86, 1.54)	0.351	0.94 (0.73, 1.21)	0.635
Calcium	Low	1.20 (0.67, 2.14)	0.548	1.09 (0.73, 1.62)	0.679	1.06 (0.74, 1.53)	0.747	1.05 (0.74, 1.50)	0.773
	High	0.74 (0.10, 5.69)	0.769	1.42 (0.34, 5.99)	0.629	–		–	
Phosphorous	Low	0.67 (0.34, 1.33)	0.251	0.85 (0.56, 1.29)	0.446	1.03 (0.71, 1.49)	0.888	1.14 (0.85, 1.53)	0.380
	High	–		–		–		–	
Magnesium	Low	0.74 (0.41, 1.32)	0.307	1.49 (0.94, 2.36)	0.089	1.40 (0.86, 2.28)	0.179	1.11 (0.73, 1.69)	0.634
Iron	Low	1.32 (0.75, 2.31)	0.338	1.09 (0.75, 1.60)	0.650	1.17 (0.81, 1.67)	0.402	1.06 (0.79, 1.42)	0.703
	High	–		1.34 (0.42, 4.30)	0.620	–		–	
Zinc	Low	1.04 (0.65, 1.67)	0.855	1.21 (0.89, 1.66)	0.228	0.90 (0.66, 1.23)	0.509	0.96 (0.74, 1.25)	0.761
	High	–		2.47 (0.59, 10.41)	0.217	–		–	
Copper	Low	1.24 (0.74, 2.06)	0.410	1.20 (0.87, 1.68)	0.270	1.25 (0.92, 1.71)	0.154	0.79 (0.60, 1.04)	0.098
	High	6.35 (0.86, 46.98)	0.070	–		–		–	
Sodium	Low	0.79 (0.37, 1.71)	0.550	1.04 (0.62, 1.72)	0.893	0.82 (0.47, 1.42)	0.475	0.96 (0.67, 1.38)	0.845
	High	0.64 (0.38, 1.09)	0.099	0.84 (0.60, 1.17)	0.303	1.08 (0.79, 1.48)	0.609	1.13 (0.85, 1.48)	0.403
Potassium	Low	0.83 (0.33, 2.08)	0.695	0.86 (0.45, 1.64)	0.646	1.18 (0.50, 2.80)	0.700	1.00 (0.43, 2.32)	0.996
Selenium	Low	1.61 (0.93, 2.77)	0.088	1.03 (0.70, 1.52	0.885	1.05 (0.76, 1.47)	0.757	1.06 (0.80, 1.39)	0.700
	High	–		–		–		–	
Caffeine	High	1.07 (0.58, 1.98)	0.834	1.63 (1.09, 2.43)	0.016*	1.59 (0.97, 2.60)	0.064	0.61 (0.37, 0.99)	0.047*
Alcohol	High	0.92 (0.49, 1.74)	0.805	1.17 (0.81, 1.74)	0.386	1.18 (0.76, 1.83)	0.465	0.72 (0.43, 1.22)	0.223
Linoleic acid	Low	0.88 (0.54, 1.43)	0.599	1.55 (1.12, 2.16)	0.009*	0.81 (0.57, 1.13)	0.216	1.16 (0.87, 1.54)	0.312
α-Linolenic acid	Low	1.12 (0.69, 1.83)	0.652	1.18 (0.86, 1.63)	0.311	0.84 (0.60, 1.16)	0.279	1.21 (0.91, 1.61)	0.193
Fish oil	Low	0.82 (0.45, 1.50)	0.522	1.70 (1.00, 2.88)	0.048*	0.86 (0.57*,* 1.30)	0.466	1.04 (0.69*,* 1.57)	0.850

RAE Retinol activity equivalents

All analyses were adjusted for age, sex, race, energy intake, educational level, marital status, employment status, smoking and study cohort except for energy, energy per weight and dietary fiber per energy which were not adjusted for energy intake

– Results are not available due to low sample sizes and mortality rate, **p* value < 0.05

**Table 11:** Associations between abnormal anthropometric measurements and mortality across levels of frailty

Anthropometric measurements		Frailty index score							
		≤ 0.1	*p* value	> 0.1 to 0.2	*p* value	> 0.2 to 0.3	*p* value	> 0.3	*p* value
		HR (95%CI)		HR (95%CI)		HR (95%CI)		HR (95%CI)	
Body mass index	Underweight	0.69 (0.09, 5.21)	0.723	0.88 (0.22, 3.61)	0.861	4.41 (2.23, 8.74)	< 0.001*	3.80 (1.60, 9.03)	0.002*
	Overweight	0.97 (0.61, 1.57)	0.915	0.88 (0.64, 1.21)	0.421	0.90 (0.65, 1.23)	0.499	0.72 (0.54, 0.98)	0.036*
	Obese	0.91 (0.52, 1.60)	0.742	0.82 (0.56, 1.19)	0.293	0.77 (0.54, 1.11)	0.161	0.89 (0.66, 1.19)	0.424
Body weight change in past 1 year	Loss > 10%	0.91 (0.41, 2.01)	0.812	1.66 (1.10, 2.50)	0.016*	1.95 (1.36, 2.79)	< 0.001*	1.61 (1.21, 2.13)	0.001*
	Gain > 10%	1.41 (0.66, 3.00)	0.380	1.66 (0.97, 2.85)	0.063	1.56 (0.98, 2.47)	0.061	1.35 (0.91, 2.01)	0.139
Waist circumference	High	1.50 (0.88, 2.56)	0.135	0.80 (0.57, 1.11)	0.185	0.77 (0.55, 1.09)	0.146	0.70 (0.50, 0.98)	0.037*
Triceps skinfold	Low	1.07 (0.53, 2.18)	0.842	1.83 (1.22, 2.74)	0.003*	2.73 (1.90, 3.94)	< 0.001*	1.36 (0.93, 2.00)	0.113
	High	1.16 (0.50, 2.71)	0.731	1.41 (0.85, 2.35)	0.184	0.74 (0.44, 1.25)	0.259	0.98 (0.64, 1.51)	0.924
Subscapular skinfold	Low	1.10 (0.50, 2.45)	0.807	1.89 (1.29, 2.77)	0.001*	1.49 (0.98, 2.26)	0.060	1.46 (0.99, 2.15)	0.058
	High	1.02 (0.41, 2.54)	0.970	0.78 (0.39, 1.54)	0.470	0.36 (0.13, 0.98)	0.046*	0.83 (0.41, 1.66)	0.589

All analyses were adjusted for age, sex, race, energy intake, educational level, marital status, employment status, smoking, and study cohort, **p* value < 0.05

**Table 12:** Associations between abnormal blood tests and mortality across levels of frailty

Blood tests		Frailty index score							
		≤ 0.1	*p* value	> 0.1 to 0.2	*p* value	> 0.2 to 0.3	*p* value	> 0.3	*p* value
		HR (95%CI)		HR (95%CI)		HR (95%CI)		HR (95%CI)	
Total lymphocyte count	Low	1.03 (0.63, 1.70)	0.908	1.61 (1.21, 2.15)	0.001*	1.26 (0.96, 1.65)	0.102	1.43 (1.14, 1.81)	0.002*
Haemoglobin	Low	0.74 (0.23, 2.36)	0.609	1.41 (0.93, 2.15)	0.110	1.33 (0.98, 1.81)	0.064	1.45 (1.13, 1.86)	0.003*
	High	0.70 (0.10, 5.09)	0.724	0.94 (0.23, 3.84)	0.934	3.04 (0.72, 12.76)	0.129	**–**	
Mean corpuscular volume	Low	1.60 (0.38, 6.64)	0.519	0.92 (0.37, 2.28)	0.863	1.72 (0.74, 3.97)	0.208	1.07 (0.56, 2.04)	0.842
	High	1.00 (0.24, 4.14)	0.999	1.19 (0.69, 2.03)	0.533	1.58 (1.02, 2.47)	0.043*	1.45 (0.99, 2.14)	0.059
Albumin	Low	2.70 (0.63, 11.62)	0.183	2.66 (1.23, 5.74)	0.013*	1.88 (0.88, 4.02)	0.105	2.51 (1.74, 3.64)	< 0.001*
Vitamin A	Low	–		–		–		**–**	
	High	0.33 (0.08, 1.34)	0.121	1.06 (0.66, 1.71)	0.795	0.74 (0.49, 1.10)	0.131	1.34 (1.02, 1.75)	0.035*
Vitamin C	Low	0.98 (0.42, 2.29)	0.958	1.80 (1.19, 2.75)	0.006*	1.25 (0.76, 2.07)	0.376	1.61 (1.13, 2.30)	0.009*
	High	0.69 (0.17, 2.83)	0.605	0.81 (3.94, 1.65)	0.554	1.02 (0.57, 1.85)	0.936	1.18 (0.72, 1.93)	0.520
Vitamin D	Low	2.01 (1.32, 3.06)	0.001*	1.45 (1.10, 1.92)	0.009*	1.62 (1.23, 2.12)	< 0.001*	1.38 (1.10, 1.73)	0.006*
	High	–		–		–		–	
Pyridoxine	Low	1.47 (0.87, 2.48)	0.151	2.11 (1.54, 2.89)	< 0.001*	1.24 (0.90, 1.71)	0.192	1.31 (1.01, 1.70)	0.04*
Folate, RBC	Low	0.92 (0.33, 2.55)	0.877	0.93 (0.43, 2.03)	0.863	1.27 (0.67, 2.41)	0.462	0.83 (0.39, 1.77)	0.630
Cobalamin	Low	1.34 (0.49, 3.68)	0.572	1.14 (0.54, 2.43)	0.728	0.68 (0.34, 1.39)	0.294	0.97 (0.57, 1.64)	0.900
α-carotene	Low	0.87 (0.46, 1.63)	0.657	1.58 (1.12, 2.23)	0.009*	1.31 (0.92, 1.85)	0.131	1.23 (0.95, 1.61)	0.121
	High	0.78 (0.40, 1.53)	0.469	0.80 (0.48, 1.32)	0.382	0.80 (0.48, 1.33)	0.383	1.00 (0.62, 1.62)	0.997
β-carotene	Low	1.04 (0.55, 1.99)	0.902	1.94 (1.33, 2.82)	0.001*	1.82 (1.26, 2.61)	0.001*	0.97 (0.71, 1.32)	0.854
	High	0.92 (0.51, 1.67)	0.784	0.91 (0.60, 1.36)	0.636	0.91 (0.63, 1.31)	0.623	0.97 (0.69, 1.36)	0.846
β-cryptoxanthin	Low	0.95 (0.49, 1.81)	0.867	1.71 (1.24, 2.36)	0.001*	1.34 (0.97, 1.84)	0.074	0.99 (0.76, 1.29)	0.951
	High	1.05 (0.62, 1.80)	0.849	0.73 (0.45, 1.17)	0.194	0.98 (0.66, 1.46)	0.916	0.94 (0.62, 1.42)	0.768
Lutein/Zeaxanthin	Low	0.73 (0.39, 1.36)	0.322	1.79 (1.33, 2.41)	< 0.001*	1.72 (1.30, 2.29)	< 0.001*	1.25 (0.99, 1.58)	0.055
	High	1.09 (0.50, 2.40)	0.822	0.96 (0.51, 1.78)	0.891	1.20 (0.63, 2.30)	0.576	1.08 (0.62, 1.88)	0.772
Lycopene	Low	1.08 (0.65, 1.79)	0.774	1.56 (1.16, 2.08)	0.003*	1.60 (1.22, 2.09)	0.001*	1.24 (0.98, 1.56)	0.075
	High	1.52 (0.77, 3.00)	0.227	1.16 (0.60, 2.22)	0.661	1.91 (1.04, 3.52)	0.037*	1.02 (0.50, 2.08)	0.965
Iron, serum	Low	0.78 (0.36, 1.69)	0.524	1.48 (0.98, 2.22)	0.061	1.41 (0.98, 2.03)	0.066	1.87 (1.46, 2.41)	< 0.001*
	High	–		0.27 (0.04, 1.90)	0.187	**–**		–	
Creatinine	Low	2.54 (1.15, 5.62)	0.021	1.01 (0.49, 2.09)	0.974	1.87 (1.02, 3.41)	0.042*	1.89 (1.05, 3.42)	0.034*
	High	2.46 (1.04, 5.78)	0.039	1.22 (0.80, 1.86)	0.363	1.04 (0.75, 1.45)	0.798	1.48 (1.15, 1.90)	0.002*
Total cholesterol	High	1.05 (0.69, 1.59)	0.824	0.95 (0.72, 1.26)	0.740	0.84 (0.64, 1.10)	0.200	0.92 (0.73, 1.16)	0.458
Triglyceride	High	1.33 (0.88, 2.02)	0.178	0.78 (0.58, 1.06)	0.116	0.95 (0.72, 1.26)	0.728	0.95 (0.75, 1.20)	0.676
HDL-c	Low	1.50 (0.67, 2.32)	0.071	1.08 (0.79, 1.46)	0.639	0.93 (0.68, 1.28)	0.673	1.18 (0.94, 1.49)	0.158
LDL-c	High	0.97 (0.51, 1.87)	0.936	0.93 (0.61, 1.41)	0.729	0.60 (0.36, 0.10)	0.050	1.20 (0.81, 1.77)	0.364
Glucose	Low	1.99 (0.27, 14.73)	0.499	1.20 (0.38, 3.78)	0.758	0.44 (0.11, 1.81)	0.256	1.49 (0.77, 2.87)	0.236
	High	1.34 (0.86, 2.09)	0.195	1.17 (0.89, 1.55)	0.263	1.09 (0.83, 1.42)	0.537	1.06 (0.85, 1.34)	0.593
Homocysteine	High	1.19 (0.16, 8.69)	0.865	1.73 (0.76, 3.90)	0.190	1.41 (0.74, 2.69)	0.298	1.71 (1.13, 2.60)	0.011*

*HDL-c* high-density lipoprotein cholesterol, *LDL-c* low-density lipoprotein cholesterol, *RBC* red blood cell

All analyses were adjusted for age, sex, race, energy intake, educational level, marital status, employment status, smoking and study cohort

– Results are not available due to low sample sizes and mortality rate, **p* value < 0.05

**Table 13:** Number of participants in each level of nutritional index score by frailty level

	Frailty index			
	≤ 0.1 *N* = 4858	> 0.1 to 0.2 *N* = 1910	> 0.2 to 0.3 *N* = 949	> 0.3 *N* = 813
Nutritional index, *N* (%) (*N* = 8530)				
≤ 0.1 (*N* = 393)	305 (7.4)	70 (4.3)	13 (1.5)	5 (0.7)
> 0.1 to 0.2 (*N* = 1967)	1435 (30.9)	354 (19.9)	110 (13.0)	68 (8.5)
> 0.2 to 0.3 (*N* = 2409)	1494 (30.5)	541 (29.2)	239 (26.8)	135 (17.8)
> 0.3 to 0.4 (*N* = 1751)	880 (17.6)	422 (21.0)	255 (25.9)	194 (24.2)
> 0.4 to 0.5 (*N* = 1203)	487 (9.0)	329 (16.9)	193 (19.3)	194 (23.5)
> 0.5 to 0.6 (*N* = 602)	209 (3.8)	139 (6.0)	104 (10.2)	150 (16.8)
> 0.6 (*N* = 205)	48 (0.9)	55 (2.6)	35 (3.2)	67 (8.4)

The percentages are weighted

**Table 14:** Association between nutritional index and mortality across levels of frailty

Nutritional index	Frailty index score							
	≤ 0.1	*p* value	> 0.1 to 0.2	*p* value	> 0.2 to 0.3	*p* value	> 0.3	*p* value
	HR (95%CI)		HR (95%CI)		HR (95%CI)		HR (95%CI)	
Nutritional index score (per 0.1 score)	1.15 (0.98, 1.35)	0.082	1.17 (1.06, 1.30)	0.002*	1.20 (1.08, 1.32)	< 0.001*	1.27 (1.16, 1.38)	< 0.001*
Nutritional index score in group								
≤ 0.2	1.00		1.00		1.00		1.00	
> 0.2 to 0.3	1.26 (0.72, 2.18)	0.420	0.99 (0.64, 1.53)	0.958	1.73 (1.01, 2.97)	0.046*	1.63 (0.91, 2.91)	0.100
> 0.3 to 0.4	1.44 (0.80, 2.59)	0.219	1.35 (0.88, 2.06)	0.164	1.80(1.06, 3.05)	0.029*	2.05 (1.20, 3.52)	0.009*
> 0.4 to 0.5	1.70 (0.88, 3.31)	0.117	2.04 (1.34, 3.11)	0.001*	2.34 (1.36, 4.01)	0.002*	1.92 (1.12, 3.31)	0.019*
> 0.5	1.58 (0.66, 3.76)	0.302	1.49 (0.89, 2.51)	0.130	2.49 (1.42, 4.38)	0.001*	3.09 (1.81, 5.27)	< 0.001*
P for trend across nutritional index group		0.097		0.001*		0.001*		< 0.001*

All analyses were adjusted for age, sex, race, educational level, marital status, employment status, smoking, and study cohort, **p* value < 0.05

**Table 15:** Combined effect of frailty and nutrition on mortality

Frailty index score	Nutrition index score	N (%)	Cox regression analysis	
			Hazard ratio (95%CI)	*p* value
≤ 0.1	≤ 0.2	1740 (24.5)	1.00 (reference)	
	> 0.2 to 0.3	1494 (19.5)	1.23 (0.71, 2.12)	0.468
	> 0.3 to 0.4	880 (11.2)	1.48 (0.84, 2.64)	0.178
	> 0.4 to 0.5	487 (5.7)	1.80 (0.95, 3.42)	0.073
	> 0.5	257 (3.0)	1.59 (0.68, 3.69)	0.281
> 0.1 to 0.2	≤ 0.2	424 (5.0)	1.59 (0.95, 2.65)	0.079
	> 0.2 to 0.3	541 (6.0)	1.59 (0.95, 2.65)	0.069
	> 0.3 to 0.4	422 (4.3)	2.13 (1.31, 3.46)	0.002*
	> 0.4 to 0.5	329 (3.5)	3.26 (2.01, 5.29)	< 0.001*
	> 0.5	194 (1.8)	2.53 (1.44, 4.45)	0.001*
> 0.2 to 0.3	≤ 0.2	123 (1.3)	1.70 (0.92, 3.15)	0.092
	> 0.2 to 0.3	239 (2.4)	2.88 (1.77, 4.68)	< 0.001*
	> 0.3 to 0.4	255 (2.3)	3.04 (1.88, 4.91)	< 0.001*
	> 0.4 to 0.5	193 (1.7)	3.97 (2.44, 6.46)	< 0.001*
	> 0.5	139 (1.2)	4.23 (2.53, 7.08)	< 0.001*
> 0.3	≤ 0.2	73 (0.6)	2.64 (1.39, 5.01)	< 0.001*
	> 0.2 to 0.3	135 (1.2)	4.38 (2.62, 7.31)	< 0.001*
	> 0.3 to 0.4	194 (1.6)	5.26 (3.29, 8.39)	< 0.001*
	> 0.4 to 0.5	194 (1.6)	5.09 (3.16, 8.18)	< 0.001*
	> 0.5	217 (1.7)	8.17 (5.16, 12.94)	< 0.001*

The percentages are weighted

All analyses were adjusted for age, sex, race, educational level, marital status, employment status, smoking, and study cohort, **p* value < 0.05

## Abbreviations

BMI: Body mass index
CI: Confidential interval
FI: Frailty index
LDL-c: Low-density lipoprotein cholesterol
NHANES: National Health and Nutrition Examination Survey
NI: Nutrition index
