# Correction to: Frailty, nutrition-related parameters, and mortality across the adult age spectrum

**DOI:** 10.1186/s12916-018-1227-z

**Published:** 2018-12-14

**Authors:** Kulapong Jayanama, Olga Theou, Joanna M. Blodgett, Leah Cahill, Kenneth Rockwood

**Affiliations:** 1 Chakri Naruebodindra Medical Institute, Faculty of Medicine Ramathibodi Hospital, Mahidol University, Bangkok, Thailand; 2 Department of Medicine, Dalhousie University, Halifax, Nova Scotia Canada; 3 Centre for Health Care of the Elderly, QEII Health Sciences Centre, Nova Scotia Health Authority, Halifax, Nova Scotia Canada; 4 MRC Unit for Lifelong Health and Ageing, UCL, London, UK; 5 Department of Nutrition, Harvard T.H. Chan School of Public Health, Boston, MA USA; 6 Division of Geriatric Medicine, Department of Medicine, Dalhousie University, Camp Hill Veterans’ Memorial Bldg., 5955 Veterans’ Memorial Lane, Halifax, Nova Scotia B3H 2E1 Canada

**Correction to: BMC Med (2018) 16:188**

**https://doi.org/10.1186/s12916-018-1176-6**

The original article [1] contained an error whereby Table 5 within the Appendix was presented incorrectly. This error has now been corrected and Table 5 is presented appropriately.

**Table 5:** Normal range of parameter

Parameter	Normal range	Score in nutritional index	
		0	1
Nutrient intakes			
Energy (kcal/day)	M ≥ 2400, F ≥ 1800	Normal range	M < 2400, F < 1800
Energy per weight (kcal/kg/day)	25-35	≥ 25	< 25
Protein (g/day)	M ≥ 56, F ≥ 46	Normal range	M < 56, F < 46
Protein per weight (g/kg/day)	< 65 years, ≥ 0.8 ≥ 65 years, ≥ 1	Normal range	< 65 years, < 0.8 ≥ 65 years, < 1.0
Carbohydrate (g/day)	≥ 180	Normal range	< 180
Simple sugar (mg/day)	M < 36, F < 25	--	--
Dietary fiber (g/1,000 kcal/day)	> 14	--	--
Percentage of fat (%)	20-35	--	--
Percentage of saturated fat (%)	< 10	Normal range	≥ 10
Cholesterol (mg/day)	< 300	--	--
Vitamin A, RAE (mcg/day)	M 900-3000 F 700-3000	Normal range	M < 900 or > 3000 F < 700 or > 3000
Vitamin C (mg/day)	M 90-2000, F 75-2000	Normal range	M < 90 or > 2000 F < 75 or > 2000
Vitamin E (mg/day)	15-1000	--	--
Vitamin K (mcg/day)	M ≥ 120, F ≥ 90	--	--
Thiamin (mg/day)	M ≥ 1.2, F ≥ 1.1	Normal range	M < 1.2, F < 1.1
Riboflavin (mg/day)	M ≥ 1.3, F ≥ 1.1	Normal range	M < 1.3, F < 1.1
Niacin (mg/day)	M 16-35, F 14-35	Normal range	M < 16 or > 35 F < 14 or > 35
Pyridoxine (mg/day)	≤ 50 years, 1.3-100 > 50 years, M 1.7-100 > 50 years, F 1.5-100	Normal range	≤ 50 years, < 1.3 or > 100 > 50 years, M < 1.7 or > 100 > 50 years, F < 1.5 or > 100
Folate (mcg/day)	400-1000	Normal range	< 400 or > 1000
Cobalamin (mcg/day)	≥ 2.4	--	--
Calcium (mg/day)	M ≤ 70 years, 1000-2500 M >70 years, 1200-2500 F ≤50 years, 1000-2500 F >50 years, 1200-2500	--	--
Phosphorous (mg/day)	700-4000	Normal range	< 700 or > 4000
Magnesium (mg/day)	M ≥ 420, F ≥ 320	--	--
Iron (mg/day)	M 8-45 F ≤ 50 years, 18-45 F > 50 years, 8-45	--	--
Zinc (mg/day)	M 11-40, F 8-40	--	--
Copper (mg/day)	0.9-10	Normal range	< 0.9 or > 10
Sodium (mg/day)	≤ 50 years, 1500-2300 > 50-70 years, 1300-2300 > 70 years, 1200-2300	≤ 50 years, ≥1,500 > 50-70 years, ≥ 1300 > 70 years, ≥1200	≤ 50 years, < 1500 > 50-70 years, < 1300 > 70 years, < 1200
Potassium (mg/day)	≥ 4700	--	--
Selenium (mcg/day)	55-400	Normal range	< 55 or > 400
Caffeine (mg/day)	≤ 400	--	--
Alcohol (g/day)	M ≤ 28, F ≤ 14	--	--
Linoleic acid (g/day)	≤ 50 years, M ≥ 17, F ≥ 12 > 50 years, M ≥ 14, F ≥ 11	--	--
α-Linolenic acid (g/day)	M ≥ 1.6, F ≥ 1.1	--	--
Fish oil (g/day)*	≥ 0.25	Normal range	< 0.25
Anthropometric measurements			
Body mass index (kg/m^2^)	18.5-24.9**	18.5-29.9	< 18.5 or ≥ 30.0
Body weight change in past 1 year (%)	≤ 10	Normal range	> 10
Waist circumference (cm)	M < 94, F < 80	Normal range	M ≥ 94, F ≥ 80
Triceps skinfold (mm)	M 7.5-24.3, F 14.0-33.7	Normal range	M < 7.5 or > 24.3 F < 14.0 or > 33.7
Subscapular skinfold (mm)	M 10.3-30.5, F 10.3-33.9	--	--
Blood tests			
Total lymphocyte count (cells/mm^3^)	> 1500	Normal range	≤ 1500
Haemoglobin (g/dL)	M 13.5-18.0, F 12.0-16.0	Normal range	M < 13.5 or > 18.0 F < 12.0 or > 16.0
MCV (fL)	80-100	Normal range	< 80 or > 100
Albumin (g/L)	35-55	Normal range	< 35 or > 55
Vitamin A (mcmol/L)	0.35-3.00	Normal range	< 0.35 or > 3.00
Vitamin C (mg/dL)	0.2-2.0	Normal range	< 0.2 or > 2.0
Vitamin D (ng/mL)	20-50	Normal range	< 20 or > 50
Pyridoxine (nmol/L)	> 20	Normal range	≤ 20
Folate, RBC (ng/mL)	≥ 140	--	--
Cobalamin (pg/L)	> 200	--	--
α-carotene (mcg/dL)	1.3-9.2	≥ 1.3	< 1.3
β-carotene (mcg/dL)	6.4-35.1	≥ 6.4	< 6.4
β-cryptoxanthin (mcg/dL)	4.0-16.4	≥ 4.0	< 4.0
Lutein/Zeaxanthin (mcg/dL)	11.1-33.0	≥ 11.1	< 11.1
Lycopene (mcg/dL)	11.9-36.1	≥ 11.9	< 11.9
Iron, serum (mcg/dL)	50-180	Normal range	< 50 or > 180
Creatinine (mg/dL)	M 0.80–1.40, F 0.56–1.00	Normal range	M < 0.80 or > 1.40 F < 0.56 or > 1.00
Total cholesterol (mg/dL)	< 200	--	--
Triglyceride (mg/dL)	< 150	Normal range	≥ 150
HDL-c (mg/dL)	M > 40, F > 50	Normal range	M ≤ 40, F ≤ 50
LDL-c (mg/dL)	< 130	--	--
Glucose (mg/dL)	70-100	Normal range	< 70 or > 100
Homocysteine (mcmol/L)	≤ 21.6	Normal range	> 21.6

*F* female; *HDL-c* High density lipoprotein cholesterol; *LDL-c* Low density lipoprotein cholesterol; *M* male; *MCV* Mean corpuscular volume; *RAE* Retinol activity equivalents; *RBC* red blood cell. -- These variables were excluded from the nutritional index due to high missing data or no relationship with high frailty; * Dietary fish oil is the combination between docosahexaenoic acid (DHA) and eicosapentaenoic acid (EPA) in dietary intake.; ** <18.5 kg/m^2^ (underweight), 25-29.9 kg/m^2^ (overweight), ≥30 kg/m^2^ (obese)
